# Crystal structure and Hirshfeld surface analysis of 4,4′-di­meth­oxy­biphenyl-3,3′,5,5′-tetra­carb­oxy­lic acid dihydrate

**DOI:** 10.1107/S2056989024002305

**Published:** 2024-03-26

**Authors:** Thomas Hanauer, Wilhelm Seichter, Monika Mazik

**Affiliations:** a Technische Universität Bergakademie Freiberg, Leipziger Str. 29, D-09596 Freiberg/Sachsen, Germany; Universidade de Sâo Paulo, Brazil

**Keywords:** biphenyl derivative, O—H⋯O hydrogen bonds, supra­molecular motifs, water cluster, helical supra­molecular strands, Hirshfeld surface, crystal structure

## Abstract

In the crystal of the title compound, C_18_H_14_O_10_·2H_2_O, the arene rings of the biphenyl moiety are tilted at an angle of 24.3 (1)°, while the planes passing through the carboxyl groups are rotated at angles of 8.6 (1) and 7.7 (1)° out of the plane of the benzene ring to which they are attached. The crystal structure is essentially stabilized by O—H⋯O bonds. Here, the carboxyl groups of neighbouring host mol­ecules are connected by cyclic *R*_2_^2^(8) synthons, leading to the formation of a three-dimensional network. The water mol­ecules in turn form helical supra­molecular strands running in the direction of the crystallographic *c*-axis (chain-like water clusters). The second H atom of each water mol­ecule provides a link to a meth­oxy O atom of the host mol­ecule. A Hirshfeld surface analysis was performed to qu­antify the contributions of the different inter­molecular inter­actions, indicating that the most important contributions to the crystal packing are from H⋯O/O⋯H (37.0%), H⋯H (26.3%), H⋯C/C⋯H (18.5%) and C⋯O/O⋯C (9.5%) inter­actions.

## Abstract 2

The crystal structure is essentially stabilized by O—H⋯O bonds. Here, the carboxyl groups of neighbouring host mol­ecules are connected by cyclic 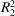 (8) synthons, leading to the formation of a three-dimensional network. The water mol­ecules in turn form helical supra­molecular strands running in the *c*-axis direction (chain-like water clusters). The second H atom of each water mol­ecule provides a link to a meth­oxy O atom of the host mol­ecule.

## 1. Chemical context

Our studies on the mol­ecular recognition of mono- and oligosaccharides with artificial receptors led to the development of various acyclic (Mazik, 2009 ▸, 2012 ▸) and macrocyclic (Lippe & Mazik, 2013 ▸, 2015 ▸; Amrhein *et al.*, 2016 ▸; Amrhein & Mazik, 2021 ▸; Leibiger *et al.*, 2022 ▸) receptor architectures. Among the acyclic compounds, those with a central aromatic core carrying three or more functionalized side arms as recognition groups have proven to be effective carbohydrate receptors. Their binding properties can be fine-tuned by varying the structural subunits of these compounds. In this context, benzene (Stapf *et al.*, 2020 ▸; Köhler *et al.*, 2020 ▸, 2021 ▸; Kaiser *et al.*, 2019 ▸), fluorene (Seidel & Mazik, 2020 ▸, 2023 ▸), di­phenyl­methane (Mazik & König, 2007 ▸; Mazik & Buthe, 2009 ▸; Koch *et al.*, 2014 ▸) or biphenyl units (Mazik & König, 2006 ▸) were used as the central aromatic platform of the receptor structures. Representatives of the last mentioned receptors can be prepared, for example, on the basis of biphenyl-3,3′,5,5′-tetra­carb­oxy­lic acid (Mazik & König, 2006 ▸). In this article we describe the crystal structure of the hydrate of 4,4′-dimeth­oxy-biphenyl-3,3′,5,5′-tetra­carb­oxy­lic acid, which is also a valuable precursor for the synthesis of receptors with a biphenyl-based scaffold. 
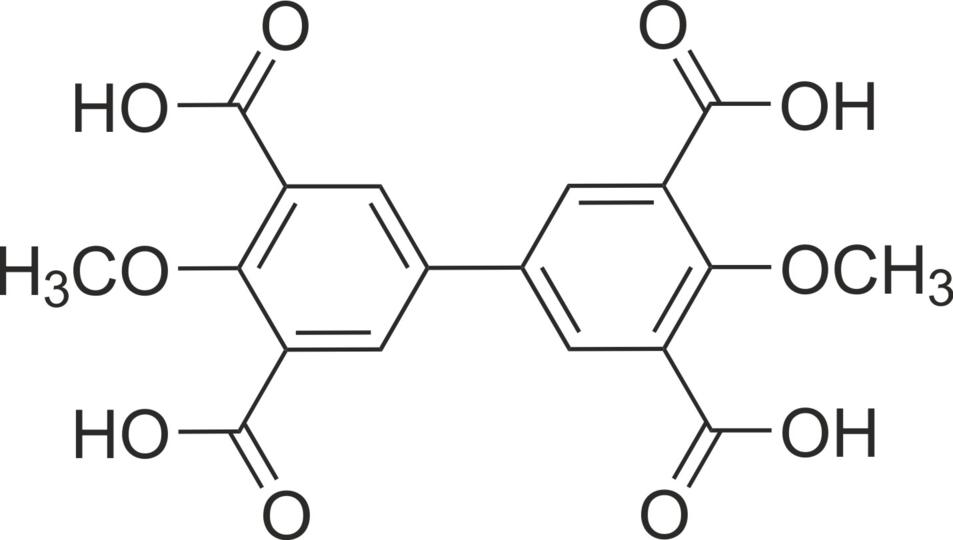


## 2. Structural commentary

**Figure 1:**
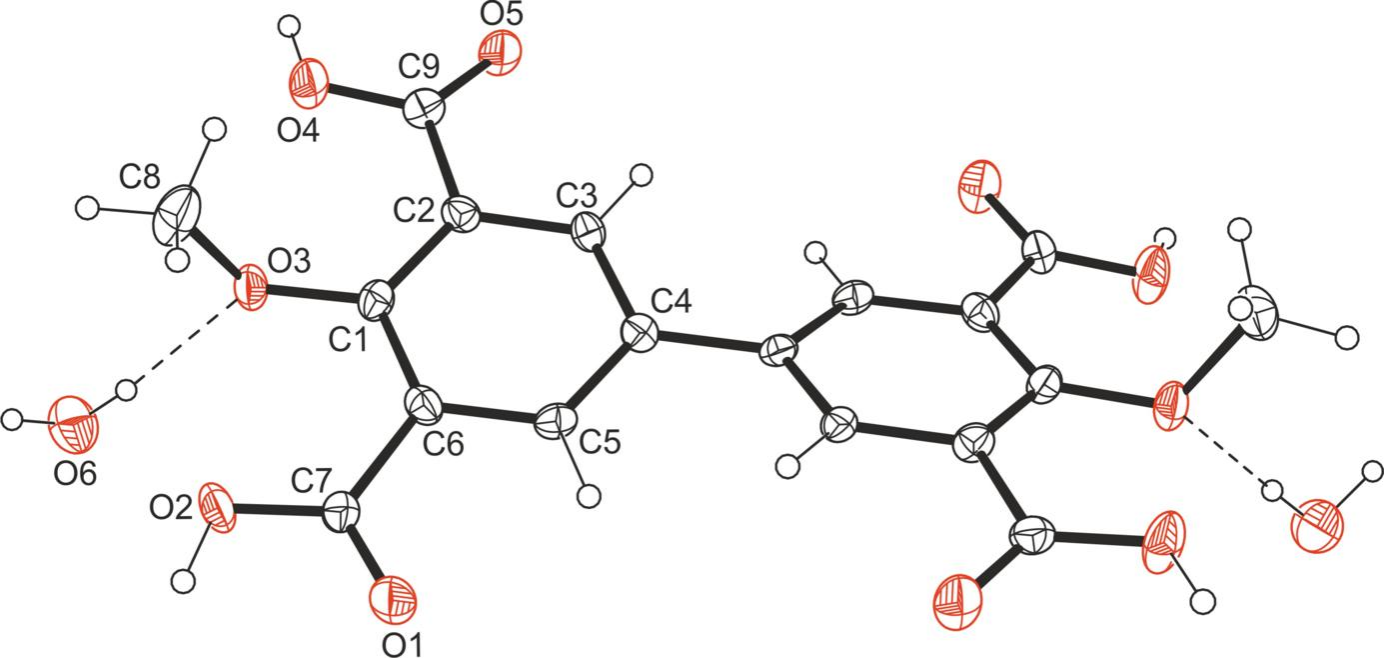
Perspective view (*ORTEP* diagram) including atom labeling of the 1:2 host–guest complex of the title mol­ecule with water. Anisotropic displacement ellipsoids are drawn at the 50% probability level.

The title compound, C_18_H_14_O_10_·2H_2_O, crystallizes in the tetra­gonal space group *I*4_1_ *cd* with one half [the second half is generated by the symmetry operation 1 − *x*, 1 − *y*, *z*] of the biphenyl-4,4′-dimeth­oxy-3,3′,5,5′-tetra­carb­oxy­lic acid (host) molecule and one water mol­ecule (guest) in the asymmetric unit of the cell. A perspective view of the 1:2 host–guest unit is shown in Fig. 1 ▸. The two benzene rings of the biphenyl moiety are twisted at an angle of 24.3 (1)°. The mean planes passing the carb­oxy groups are inclined at angles of 8.6 (1) and 7.7 (1)° with respect to the planes of the respective benzene rings. The bond lengths within the host mol­ecule resemble those found in the crystal structure of biphenyl-3,3′,5,5′-tetra­carb­oxy­lic acid (Coles *et al.*, 2002 ▸).

## 3. Supra­molecular features and Hirshfeld surface analysis

**Figure 2:**
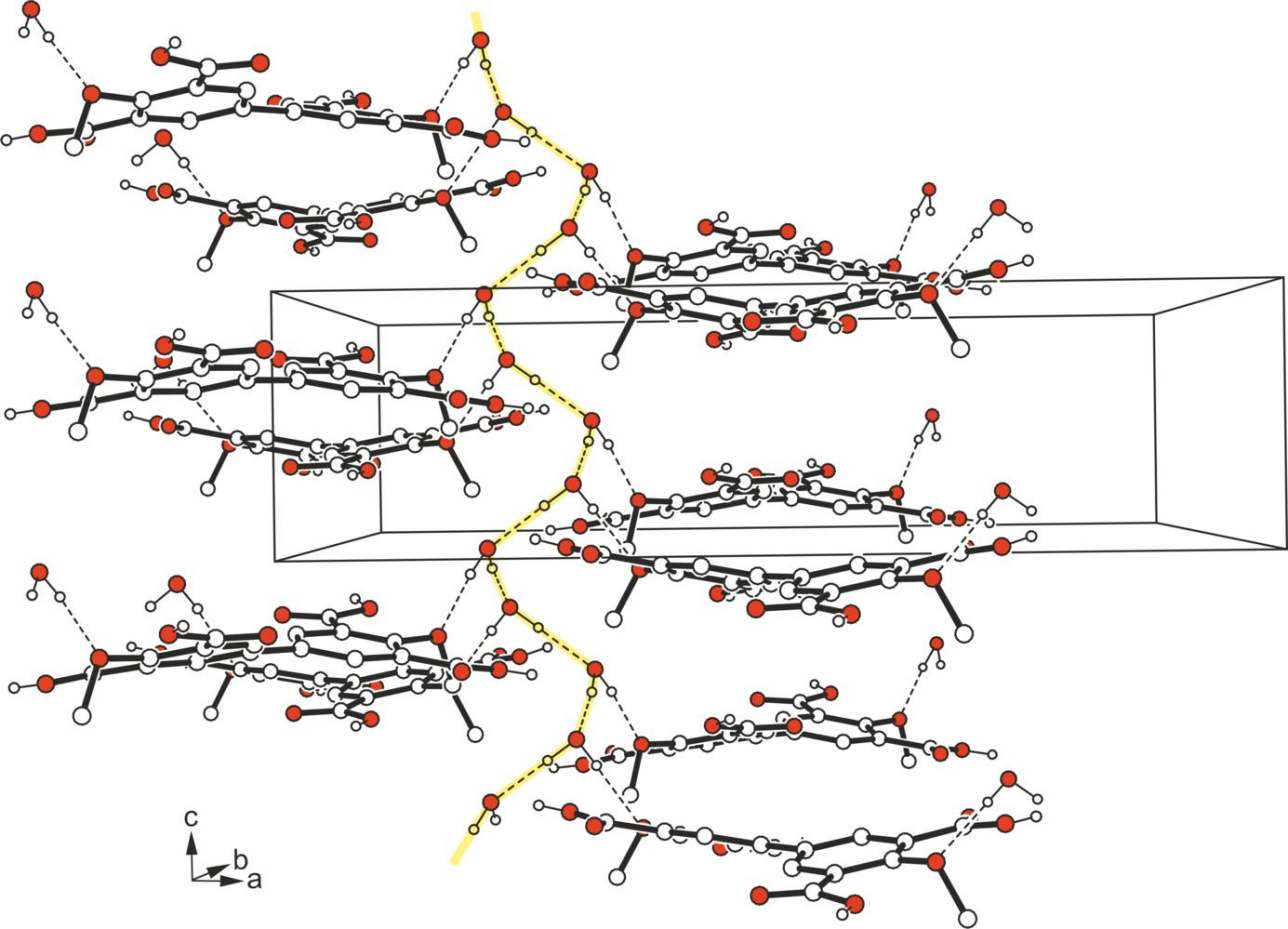
Part of the crystal structure of the title compound showing the helical strands of O—H⋯O-bonded water mol­ecules running along the *c*-axis direction. Dashed lines represent hydrogen-bond inter­actions.

**Figure 3:**
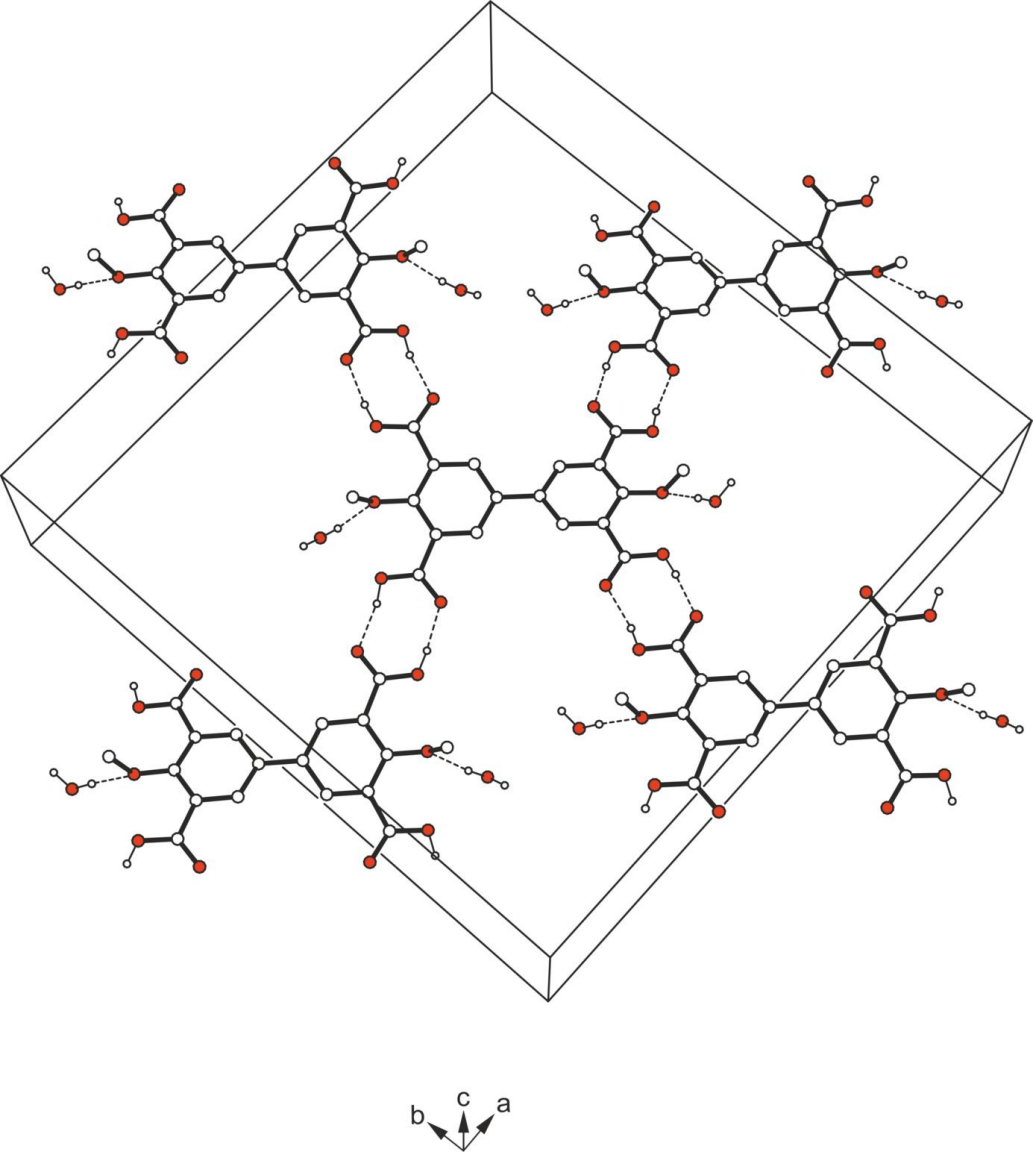
Part of the crystal structure of the title compound showing the mode of hydrogen bonding.

**Figure 4:**
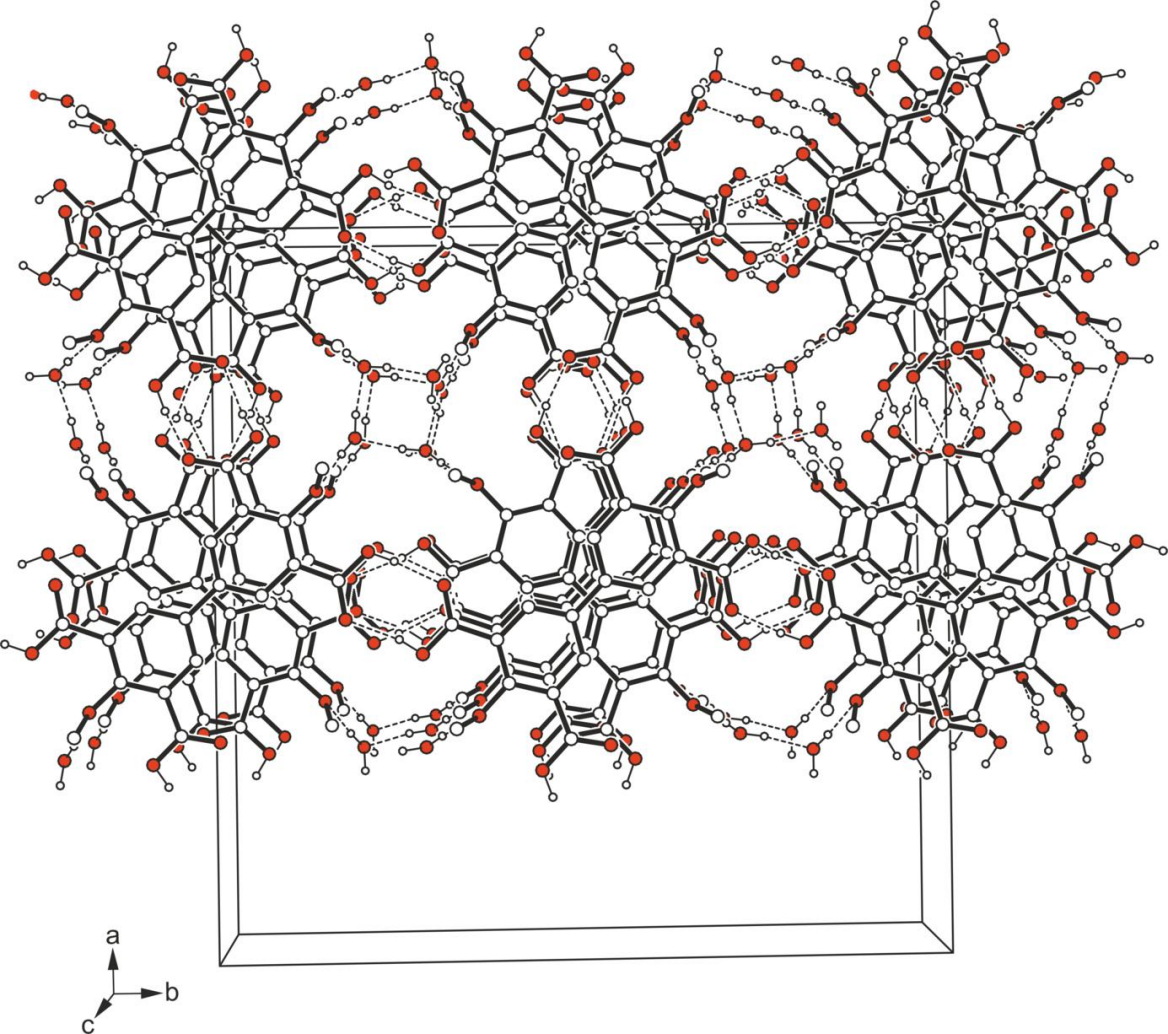
Packing diagram of the title compound viewed down the crystallographic *c*-axis. Dashed lines represent hydrogen-bond inter­actions.

The crystal structure is mainly stabilized by O—H⋯O bonds (Table 1 ▸). On the one hand, these hydrogen bonds contribute to the connection of the host mol­ecules *via* cyclic synthons of the structure 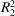 (8) (Etter *et al.*, 1990 ▸; Etter, 1991 ▸; Bernstein *et al.*, 1995 ▸), thus creating a three-dimensional cross-linking of these mol­ecules. On the other hand, the water mol­ecules in turn form infinite helical strands running in the *c*-axis direction (Fig. 2 ▸). The arrangement of the water mol­ecules in this helical structure corresponds to the fourfold symmetry element (4_1_ axis) of the crystal structure. The second H atom of the water mol­ecule serves as a binding site for a hydrogen bond to the O atom of the meth­oxy group (Fig. 3 ▸). Taking the inter­actions between the water mol­ecules into account, their arrangement could also be described as water clusters, which belong to the class of infinite chains (for nomenclature of water clusters, see: Infantes & Motherwell, 2002 ▸; Mascal *et al.*, 2006 ▸; for examples of other water clusters reported by our group, see: Rosin *et al.*, 2017 ▸). Furthermore, the H⋯*Cg* distances of 2.96 and 2.99 Å involving the hydrogen atoms H3 and H5 (symmetry operations: *x*, 1 − *y*, 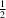 + *z*; 1 − *x*, *y*, − 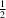 + *z*) indicate the presence of weak C—H⋯π inter­actions. A packing diagram of the title compound viewed along the *c*-axis direction is presented in Fig. 4 ▸.

**Figure 5:**
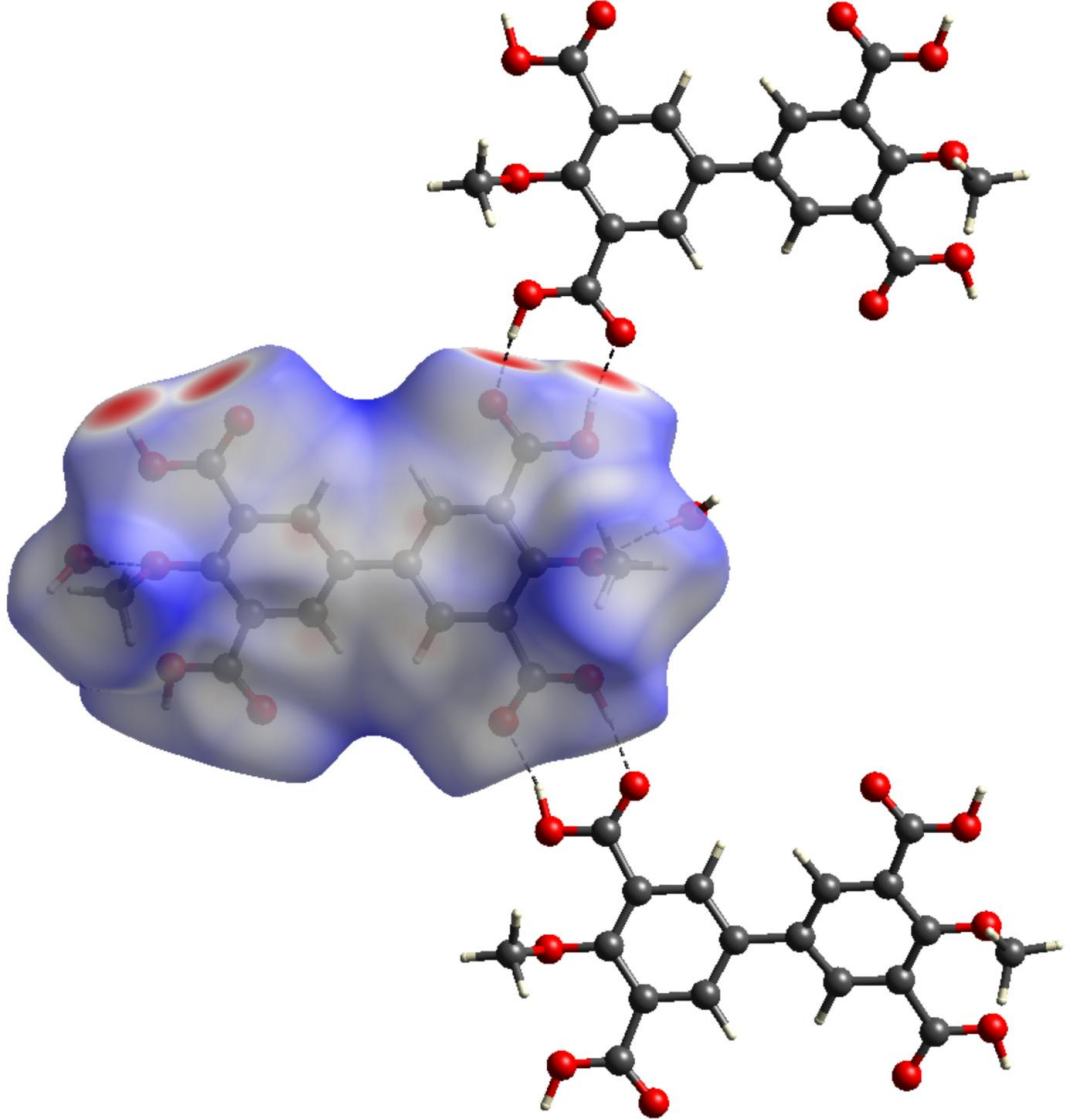
View of the three-dimensional Hirshfeld surface of the title compound, plotted over *d*_norm_ in the range −0.7606 to 1.5689 a.u., generated with *CrystalExplorer* (Spackman *et al.*, 2021 ▸)

**Figure 6:**
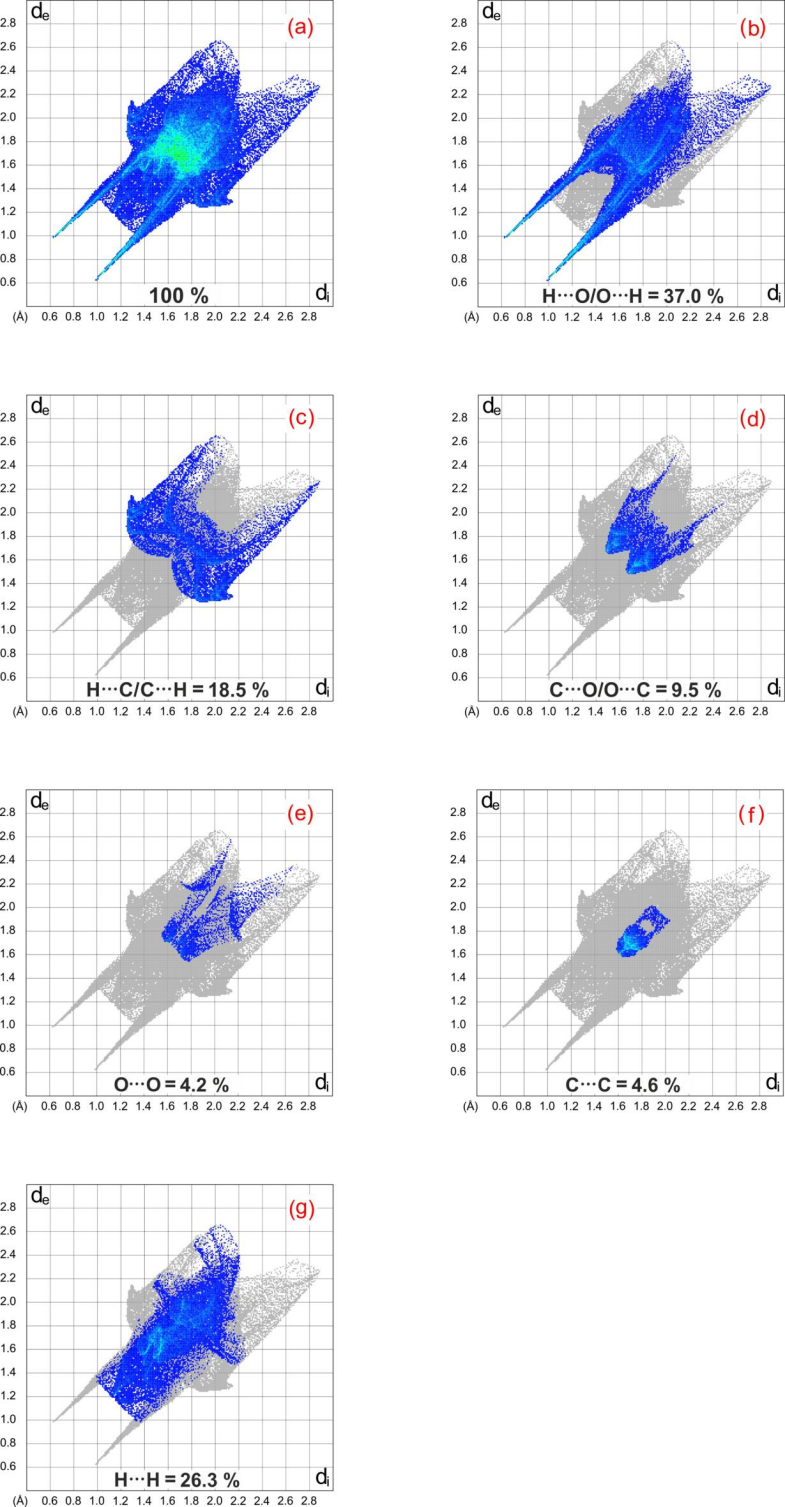
Two-dimensional fingerprint plots of the title compound, showing (*a*) all inter­actions, and delineated into (*b*) H⋯O/O⋯H, (*c*) H⋯C/C⋯H, (*d*) C⋯O/O⋯C, (*e*) O⋯O, (*f*) C⋯C and (*g*) H⋯H inter­actions. The *d*_i_ and *d*_e_ values represent the closest inter­nal and external distances (in Å) from given points on the Hirshfeld surface.

In order to visualize and qu­antify inter­molecular inter­actions a Hirshfeld surface analysis (Spackman & Byrom, 1997 ▸; McKinnon *et al.*, 1998 ▸) was performed using *CrystalExplorer* (Version 21.5, Spackman *et al.*, 2021 ▸). The Hirshfeld surface mapped over *d*_norm_ using a standard surface resolution with a fixed colour scale of −0.7603 to 1.5689 a.u. is shown in Fig. 5 ▸. The red spots on the *d*_norm_ surface represent O—H⋯O hydrogen bonds. The full two-dimensional fingerprint plots and those delineated into different types of inter­actions (McKinnon, 2007 ▸) are illustrated in Fig. 6 ▸. They reveal that H⋯O/O⋯H contacts (Fig. 6 ▸ *b*), *i.e.* strong hydrogen bonds, contribute 37.0% of the Hirshfeld surface. The two weakly pronounced wings in the fingerprint plot prove the presence of inter­molecular inter­actions of the C—H⋯π type. H⋯H contacts represent 26.3% of the fingerprint plot, while H⋯C/C⋯H and C⋯O/O⋯C contacts cover 18.5% and 9.5% of the Hirshfeld surface, respectively.

## 4. Database survey

A search in the Cambridge Structural Database (CSD, Version 5.44, update April 2023; Groom *et al.*, 2016 ▸) for 4,4′-disubstituted derivatives of biphenyl-3,3′,5,5′-tetra­carb­oxy­lic acid gave no hits; however, the crystal structure of biphenyl-3,3′,5,5′-tetra­carb­oxy­lic acid is known (refcode: PUYTEI; Coles *et al.*, 2002 ▸). A comparison between the structure of the title compound and the unsolvated structure of biphenyl-3,3′,5,5′-tetra­carb­oxy­lic acid (PUYTEI) provides a hint about the influence of the water mol­ecules on the packing of the crystal structure. The difference in the space-group symmetries (*I*4_1_ *cd vs P*2_1_/*c*) suggests structural differences. In the structure of PUYTEI, a supra­molecular arrangement of three inter­penetrating corrugated layers forms structure domains that extend parallel to the crystallographic *ac* plane. Within a given corrugated layer, adjacent mol­ecules are also linked *via* their carboxyl groups through eight-membered ring synthons. Furthermore, the mol­ecules within the structure domains are arranged such that offset π–π-stacking inter­actions [*d*(*Cg*⋯*Cg*) = 3.636 Å] are effective between their aromatic units.

## 5. Synthesis and crystallization

To a mixture of 760 mg (1.51 mmol) of 4,4′-dimeth­oxy-3,3′,5,5′-bi­phenyl­tetra­carb­oxy­lic acid tetra­ethyl ester and 60 mL of water, 1.01 g (17.9 mmol) of potassium hydroxide were added. After heating to boiling point for up to 18 h, the solution was cooled and acidified with semi-concentrated sulfuric acid. The white solid was filtered and dried under reduced pressure. Yield 96% (578 mg, 1.48 mmol); m.p. 528 K. ^1^H NMR (500 MHz, DMSO-*d*_6_, ppm): δ = 3.85 (*s*, 6H), 8.08 (*s*, 4H), 13.31 (*br. s*, 4H). ^13^C NMR (125 MHz, DMSO-*d*_6_, ppm): δ = 63.0, 128.4, 131.0, 133.1, 157.1, 166.7. Single crystals suitable for X-ray analysis were obtained by recrystallizing the resulting solid from water.

## 6. Refinement

Crystal data, data collection and structure refinement details are summarized in Table 2 ▸. The non-hydrogen atoms were refined anisotropically. The carboxyl H atoms and the hydrogen atoms of the water mol­ecules were identified in difference-Fourier maps and their *U*_iso_ parameters refined freely. All other H atoms were placed in calculated positions and refined using a riding model with C—H = 0.95–0.98 Å and *U*_iso_(H) = 1.2 or 1.5 *U*_eq_(C).

## Supplementary Material

Crystal structure: contains datablock(s) I. DOI: 10.1107/S2056989024002305/ex2080sup1.cif

Structure factors: contains datablock(s) I. DOI: 10.1107/S2056989024002305/ex2080Isup2.hkl

CCDC reference: 2339048

Additional supporting information:  crystallographic information; 3D view; checkCIF report

## Figures and Tables

**Table 1:** Hydrogen-bond geometry (Å, °)

*D*—H⋯*A*	*D*—H	H⋯*A*	*D*⋯*A*	*D*—H⋯*A*
O2—H1*O*2⋯O5^i^	0.88 (6)	1.80 (6)	2.663 (4)	166 (7)
O4—H1*O*4⋯O1^ii^	0.88 (3)	1.74 (3)	2.611 (4)	174 (7)
O6—H1*O*6⋯O6^iii^	0.91 (3)	1.86 (3)	2.768 (4)	173 (5)
O6—H2*O*6⋯O3	0.90 (3)	2.04 (3)	2.941 (5)	174 (7)

Symmetry codes: (i) 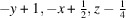 ; (ii) 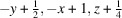 ; (iii) 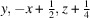 .

**Table 2:** Experimental details

Crystal data	
Chemical formula	C_18_H_14_O_10_·2H_2_O
*M*_r_	426.32
Crystal system, space group	Tetragonal, *I*4_1_ *c* *d*
Temperature (K)	123
*a*, *c* (Å)	24.069 (3), 6.4468 (8)
*V* (Å^3^)	3734.6 (9)
*Z*	8
Radiation type	Mo *K*α
μ (mm^−1^)	0.13
Crystal size (mm)	0.40 × 0.03 × 0.03

Data collection	
Diffractometer	Stoe IPDS 2T
No. of measured, independent and observed [*I* > 2σ(*I*)] reflections	14573, 1914, 1502
*R*_int_	0.055
(sin θ/λ)_max_ (Å^−1^)	0.627

Refinement	
*R*[*F* ^2^ > 2σ(*F* ^2^)], *wR*(*F* ^2^), *S*	0.039, 0.093, 1.08
No. of reflections	1914
No. of parameters	153
No. of restraints	4
H-atom treatment	H atoms treated by a mixture of independent and constrained refinement
Δρ_max_, Δρ_min_ (e Å^−3^)	0.22, −0.20
Absolute structure	Flack *x* determined using 575 quotients [(*I* ^+^)−(*I* ^−^)]/[(*I* ^+^)+(*I* ^−^)] (Parsons *et al.*, 2013 ▸)
Absolute structure parameter	−2.2 (10)

Computer programs: *X-AREA* and *X-RED* (Stoe & Cie, 2002 ▸), *SHELXT2018/2* (Sheldrick, 2015 ▸a), *SHELXL2018/3* (Sheldrick, 2015 ▸b), *XP* in *SHELXTL* (Sheldrick, 2008 ▸), *WinGX* (Farrugia, 2012 ▸), *publCIF* (Westrip, 2010 ▸) and *ShelXle* (Hübschle *et al.*, 2011 ▸).

## supplementary crystallographic information

### Crystal data

**Table:**

C_18_H_14_O_10_·2H_2_O	*D*_x_ = 1.516 Mg m^−^^3^
*M**_r_* = 426.32	Mo *K*α radiation, λ = 0.71073 Å
Tetragonal, *I*4_1_*c**d*	Cell parameters from 1164 reflections
*a* = 24.069 (3) Å	θ = 3.7–23.3°
*c* = 6.4468 (8) Å	µ = 0.13 mm^−^^1^
*V* = 3734.6 (9) Å^3^	*T* = 123 K
*Z* = 8	Needle, colorless
*F*(000) = 1776	0.40 × 0.03 × 0.03 mm

### Data collection

**Table:**

Stoe IPDS 2T diffractometer	1502 reflections with *I* > 2σ(*I*)
Radiation source: sealed X-ray tube, 12 x 0.4 mm long-fine focus	*R*_int_ = 0.055
Plane graphite monochromator	θ_max_ = 26.5°, θ_min_ = 2.7°
Detector resolution: 6.67 pixels mm^-1^	*h* = −30→30
rotation method scans	*k* = −30→30
14573 measured reflections	*l* = −7→8
1914 independent reflections	

### Refinement

**Table:**

Refinement on *F*^2^	Hydrogen site location: mixed
Least-squares matrix: full	H atoms treated by a mixture of independent and constrained refinement
*R*[*F*^2^ > 2σ(*F*^2^)] = 0.039	*w* = 1/[σ^2^(*F*_o_^2^) + (0.0377*P*)^2^ + 2.9912*P*] where *P* = (*F*_o_^2^ + 2*F*_c_^2^)/3
*wR*(*F*^2^) = 0.093	(Δ/σ)_max_ < 0.001
*S* = 1.08	Δρ_max_ = 0.22 e Å^−^^3^
1914 reflections	Δρ_min_ = −0.20 e Å^−^^3^
153 parameters	Absolute structure: Flack *x* determined using 575 quotients [(*I*^+^)-(*I*^-^)]/[(*I*^+^)+(*I*^-^)] (Parsons *et al.*, 2013)
4 restraints	Absolute structure parameter: −2.2 (10)

### Special details

**Table:**

Geometry. All esds (except the esd in the dihedral angle between two l.s. planes) are estimated using the full covariance matrix. The cell esds are taken into account individually in the estimation of esds in distances, angles and torsion angles; correlations between esds in cell parameters are only used when they are defined by crystal symmetry. An approximate (isotropic) treatment of cell esds is used for estimating esds involving l.s. planes.

### Fractional atomic coordinates and isotropic or equivalent isotropic displacement parameters (Å^2^)

**Table:**

	*x*	*y*	*z*	*U*_iso_*/*U*_eq_	
O1	0.51833 (11)	0.31455 (13)	0.2339 (6)	0.0237 (8)	
O2	0.42995 (12)	0.28601 (12)	0.2186 (6)	0.0239 (8)	
H1O2	0.449 (3)	0.256 (3)	0.185 (12)	0.05 (2)*	
O3	0.35137 (11)	0.35799 (11)	0.3165 (6)	0.0179 (5)	
O4	0.28338 (12)	0.43760 (11)	0.4265 (6)	0.0248 (8)	
H1O4	0.2495 (15)	0.450 (3)	0.443 (13)	0.07 (2)*	
O5	0.31378 (12)	0.52496 (11)	0.4093 (6)	0.0222 (7)	
C1	0.39269 (17)	0.39659 (18)	0.3264 (9)	0.0140 (6)	
C2	0.37996 (17)	0.45289 (16)	0.3673 (7)	0.0140 (9)	
C3	0.42244 (16)	0.49250 (17)	0.3661 (6)	0.0127 (9)	
H3	0.413302	0.530267	0.392110	0.015*	
C4	0.47773 (17)	0.47854 (17)	0.3282 (9)	0.0124 (5)	
C5	0.48953 (17)	0.42264 (17)	0.2878 (7)	0.0137 (9)	
H5	0.526771	0.412055	0.259073	0.016*	
C6	0.44816 (17)	0.38202 (16)	0.2884 (7)	0.0142 (9)	
C7	0.46785 (15)	0.32382 (17)	0.2435 (7)	0.0156 (9)	
C8	0.33949 (17)	0.32976 (16)	0.5113 (7)	0.0271 (9)	
H8A	0.308463	0.303926	0.491492	0.041*	
H8B	0.329565	0.357244	0.617154	0.041*	
H8C	0.372435	0.309100	0.556346	0.041*	
C9	0.32219 (16)	0.47463 (15)	0.4027 (8)	0.0155 (9)	
O6	0.30431 (14)	0.28767 (14)	−0.0127 (6)	0.0401 (8)	
H1O6	0.302 (2)	0.2570 (16)	0.070 (7)	0.042 (15)*	
H2O6	0.321 (3)	0.309 (3)	0.084 (9)	0.09 (3)*	

### Atomic displacement parameters (Å^2^)

**Table:**

	*U*^11^	*U*^22^	*U*^33^	*U*^12^	*U*^13^	*U*^23^
O1	0.0149 (13)	0.0148 (15)	0.041 (2)	0.0036 (10)	0.0022 (14)	−0.0036 (15)
O2	0.0179 (13)	0.0110 (15)	0.043 (2)	−0.0020 (11)	0.0022 (14)	−0.0074 (15)
O3	0.0136 (13)	0.0131 (13)	0.0270 (12)	−0.0032 (9)	0.0003 (12)	−0.0021 (12)
O4	0.0101 (14)	0.0168 (14)	0.047 (2)	0.0004 (10)	0.0038 (15)	−0.0043 (14)
O5	0.0126 (13)	0.0170 (13)	0.037 (2)	0.0036 (10)	0.0038 (15)	−0.0010 (14)
C1	0.0113 (19)	0.0159 (19)	0.0146 (14)	−0.0022 (11)	−0.0012 (17)	0.0004 (17)
C2	0.0126 (19)	0.0133 (18)	0.016 (2)	0.0019 (15)	−0.0009 (17)	−0.0020 (17)
C3	0.0131 (17)	0.0119 (18)	0.013 (2)	−0.0007 (14)	−0.0030 (18)	−0.0004 (17)
C4	0.0117 (19)	0.0137 (19)	0.0118 (14)	0.0007 (10)	−0.0030 (16)	−0.0001 (17)
C5	0.0093 (17)	0.0163 (19)	0.016 (2)	0.0034 (14)	−0.0002 (17)	−0.0001 (19)
C6	0.019 (2)	0.0106 (19)	0.014 (2)	−0.0002 (15)	0.0004 (18)	−0.0002 (17)
C7	0.0157 (17)	0.0125 (19)	0.019 (2)	−0.0012 (15)	0.0031 (19)	0.0006 (17)
C8	0.022 (2)	0.024 (2)	0.035 (2)	−0.0026 (16)	0.0039 (18)	0.0104 (18)
C9	0.0138 (19)	0.0166 (18)	0.016 (2)	0.0024 (15)	−0.0012 (18)	0.0016 (17)
O6	0.045 (2)	0.0366 (18)	0.039 (2)	−0.0051 (15)	−0.0063 (16)	−0.0029 (16)

### Geometric parameters (Å, º)

**Table:**

O1—C7	1.237 (4)	C3—C4	1.394 (6)
O2—C7	1.298 (5)	C3—H3	0.9500
O2—H1O2	0.88 (6)	C4—C5	1.400 (6)
O3—C1	1.363 (4)	C4—C4^i^	1.489 (6)
O3—C8	1.456 (5)	C5—C6	1.396 (6)
O4—C9	1.300 (5)	C5—H5	0.9500
O4—H1O4	0.88 (3)	C6—C7	1.507 (6)
O5—C9	1.229 (4)	C8—H8A	0.9800
C1—C6	1.402 (6)	C8—H8B	0.9800
C1—C2	1.414 (6)	C8—H8C	0.9800
C2—C3	1.398 (6)	O6—H1O6	0.91 (3)
C2—C9	1.503 (6)	O6—H2O6	0.90 (3)
			
C7—O2—H1O2	105 (4)	C4—C5—H5	119.1
C1—O3—C8	114.9 (4)	C5—C6—C1	120.3 (4)
C9—O4—H1O4	117 (5)	C5—C6—C7	115.2 (4)
O3—C1—C6	121.1 (4)	C1—C6—C7	124.5 (3)
O3—C1—C2	120.3 (4)	O1—C7—O2	123.9 (4)
C6—C1—C2	118.6 (2)	O1—C7—C6	119.1 (4)
C3—C2—C1	119.6 (4)	O2—C7—C6	117.0 (3)
C3—C2—C9	116.1 (3)	O3—C8—H8A	109.5
C1—C2—C9	124.2 (3)	O3—C8—H8B	109.5
C4—C3—C2	122.3 (4)	H8A—C8—H8B	109.5
C4—C3—H3	118.8	O3—C8—H8C	109.5
C2—C3—H3	118.8	H8A—C8—H8C	109.5
C3—C4—C5	117.3 (2)	H8B—C8—H8C	109.5
C3—C4—C4^i^	121.3 (5)	O5—C9—O4	123.6 (4)
C5—C4—C4^i^	121.4 (5)	O5—C9—C2	120.1 (4)
C6—C5—C4	121.9 (4)	O4—C9—C2	116.4 (3)
C6—C5—H5	119.1	H1O6—O6—H2O6	95 (6)
			
C8—O3—C1—C6	90.3 (6)	C4—C5—C6—C7	−179.6 (5)
C8—O3—C1—C2	−92.3 (6)	O3—C1—C6—C5	176.4 (4)
O3—C1—C2—C3	−176.6 (5)	C2—C1—C6—C5	−1.1 (9)
C6—C1—C2—C3	0.9 (9)	O3—C1—C6—C7	−2.7 (8)
O3—C1—C2—C9	0.1 (8)	C2—C1—C6—C7	179.8 (4)
C6—C1—C2—C9	177.6 (4)	C5—C6—C7—O1	8.4 (7)
C1—C2—C3—C4	−0.8 (8)	C1—C6—C7—O1	−172.4 (5)
C9—C2—C3—C4	−177.8 (4)	C5—C6—C7—O2	−172.1 (4)
C2—C3—C4—C5	0.9 (8)	C1—C6—C7—O2	7.1 (7)
C2—C3—C4—C4^i^	−179.9 (3)	C3—C2—C9—O5	6.7 (7)
C3—C4—C5—C6	−1.1 (8)	C1—C2—C9—O5	−170.1 (5)
C4^i^—C4—C5—C6	179.7 (3)	C3—C2—C9—O4	−172.8 (4)
C4—C5—C6—C1	1.2 (8)	C1—C2—C9—O4	10.4 (7)

Symmetry code: (i) −*x*+1, −*y*+1, *z*.

### Hydrogen-bond geometry (Å, º)

**Table:**

*D*—H···*A*	*D*—H	H···*A*	*D*···*A*	*D*—H···*A*
O2—H1*O*2···O5^ii^	0.88 (6)	1.80 (6)	2.663 (4)	166 (7)
O4—H1*O*4···O1^iii^	0.88 (3)	1.74 (3)	2.611 (4)	174 (7)
O6—H1*O*6···O6^iv^	0.91 (3)	1.86 (3)	2.768 (4)	173 (5)
O6—H2*O*6···O3	0.90 (3)	2.04 (3)	2.941 (5)	174 (7)

Symmetry codes: (ii) −*y*+1, −*x*+1/2, *z*−1/4; (iii) −*y*+1/2, −*x*+1, *z*+1/4; (iv) *y*, −*x*+1/2, *z*+1/4.

## References

[bb1] Amrhein, F., Lippe, J. & Mazik, M. (2016). *Org. Biomol. Chem.* **14**, 10648–10659. 10.1039/c6ob01682k 27782281

[bb2] Amrhein, F. & Mazik, M. (2021). *Eur. J. Org. Chem.* pp. 6282–6303.

[bb3] Bernstein, J., Davis, R. E., Shimoni, L. & Chang, N.-L. (1995). *Angew. Chem. Int. Ed. Engl.* **34**, 1555–1573.

[bb4] Coles, S. J., Holmes, R., Hursthouse, M. B. & Price, D. J. (2002). *Acta Cryst.* E**58**, o626–o628.

[bb5] Etter, M. C. (1991). *J. Phys. Chem.* **95**, 4601–4610.

[bb6] Etter, M. C., MacDonald, J. C. & Bernstein, J. (1990). *Acta Cryst.* B**46**, 256–262. 10.1107/s0108768189012929 2344397

[bb7] Farrugia, L. J. (2012). *J. Appl. Cryst.* **45**, 849–854.

[bb8] Groom, C. R., Bruno, I. J., Lightfoot, M. P. & Ward, S. C. (2016). *Acta Cryst.* B**72**, 171–179. 10.1107/S2052520616003954 PMC4822653 27048719

[bb9] Hübschle, C. B., Sheldrick, G. M. & Dittrich, B. (2011). *J. Appl. Cryst.* **44**, 1281–1284. 10.1107/S0021889811043202 PMC3246833 22477785

[bb10] Infantes, L. & Motherwell, S. (2002). *CrystEngComm*, **4**, 454–461.

[bb11] Kaiser, S., Geffert, C. & Mazik, M. (2019). *Eur. J. Org. Chem.* pp. 7555–7562.

[bb12] Koch, N., Rosien, J.-R. & Mazik, M. (2014). *Tetrahedron*, **70**, 8758–8767.

[bb13] Köhler, L., Hübler, C., Seichter, W. & Mazik, M. (2021). *RSC Adv.* **11**, 22221–22229. 10.1039/d1ra03390e PMC9034237 35480817

[bb14] Köhler, L., Seichter, W. & Mazik, M. (2020). *Eur. J. Org. Chem.* pp. 7023–7034.

[bb15] Leibiger, B., Stapf, M. & Mazik, M. (2022). *Molecules*, **27**, 7630–7645. 10.3390/molecules27217630 PMC9654292 36364458

[bb16] Lippe, J. & Mazik, M. (2013). *J. Org. Chem.* **78**, 9013–9020. 10.1021/jo400933q 24000949

[bb17] Lippe, J. & Mazik, M. (2015). *J. Org. Chem.* **80**, 1427–1439. 10.1021/jo502335u 25531805

[bb18] Mascal, M., Infantes, L. & Chisholm, J. (2006). *Angew. Chem. Int. Ed.* **45**, 32–36. 10.1002/anie.200501839 16307459

[bb19] Mazik, M. (2009). *Chem. Soc. Rev.* **38**, 935–956. 10.1039/b710910p 19421573

[bb20] Mazik, M. (2012). *RSC Adv.* **2**, 2630–2642.

[bb21] Mazik, M. & Buthe, A. (2009). *Org. Biomol. Chem.* **7**, 2063–2071. 10.1039/b901173k 19421443

[bb22] Mazik, M. & König, A. (2006). *J. Org. Chem.* **71**, 7854–7857. 10.1021/jo0610309 16995697

[bb23] Mazik, M. & König, A. (2007). *Eur. J. Org. Chem.* pp. 3271–3276.

[bb24] McKinnon, J. J., Jayatilaka, D. & Spackman, M. A. (2007). *Chem. Commun.* pp. 3814–3816. 10.1039/b704980c 18217656

[bb25] McKinnon, J. J., Mitchell, A. S. & Spackman, M. A. (1998). *Chem. Eur. J.* **4**, 2136–2141.

[bb26] Parsons, S., Flack, H. D. & Wagner, T. (2013). *Acta Cryst.* B**69**, 249–259. 10.1107/S2052519213010014 PMC3661305 23719469

[bb27] Rosin, R., Seichter, W., Schwarzer, A. & Mazik, M. (2017). *Eur. J. Org. Chem.* pp. 6038–6051.

[bb28] Sheldrick, G. M. (2008). *Acta Cryst.* A**64**, 112–122. 10.1107/S0108767307043930 18156677

[bb29] Sheldrick, G. M. (2015*a*). *Acta Cryst.* A**71**, 3–8.

[bb30] Sheldrick, G. M. (2015*b*). *Acta Cryst.* C**71**, 3–8.

[bb31] Seidel, P. & Mazik, M. (2020). *ChemistryOpen*, **9**, 1202-1213. 10.1002/open.202000268 PMC7689417 33304735

[bb32] Seidel, P. & Mazik, M. (2023). *ChemistryOpen*, **12**, e202300019. 10.1002/open.202300019 PMC10344870 37442791

[bb33] Spackman, P. R. & Byrom, P. G. (1997). *Chem. Phys. Lett.* **267**, 215–220.

[bb34] Spackman, P. R., Turner, M. J., McKinnon, J. J., Wolff, S. K., Grimwood, D. J., Jayatilaka, D. & Spackman, M. A. (2021). *J. Appl. Cryst.* **54**, 1006–1011. 10.1107/S1600576721002910 PMC8202033 34188619

[bb35] Stapf, M., Seichter, W. & Mazik, M. (2020). *Eur. J. Org. Chem.* pp. 4900–4915.

[bb36] Stoe & Cie (2002). *X-RED* and *X-AREA*. Stoe & Cie, Darmstadt, Germany.

[bb37] Westrip, S. P. (2010). *J. Appl. Cryst.* **43**, 920–925.

